# Interleaved ^**23**^Na/ ^1^H MRI of the human heart at 7 T using a combined ^23^Na/ ^1^H coil setup and ^1^H parallel transmission

**DOI:** 10.1002/mrm.30426

**Published:** 2025-03-10

**Authors:** Laurent Ruck, Nico Egger, Tobias Wilferth, Judith Schirmer, Lena Vanessa Gast, Sophia Nagelstraßer, Saskia Wildenberg, Andreas Bitz, Titus Lanz, Tanja Platt, Simon Konstandin, Christoph Kopp, Michael Uder, Armin Michael Nagel

**Affiliations:** ^1^ Institute of Radiology, University Hospital Erlangen Friedrich‐Alexander‐Universität Erlangen–Nürnberg Erlangen Germany; ^2^ Electrical Engineering and Information Technology University of Applied Sciences – FH Aachen Aachen Germany; ^3^ Rapid Biomedical GmbH Rimpar Germany; ^4^ Division of Medical Physics in Radiology German Cancer Research Centre (DKFZ) Heidelberg Germany; ^5^ Fraunhofer Institute for Digital Medicine MEVIS Bremen Germany; ^6^ Department of Nephrology and Hypertension Friedrich‐Alexander‐Universität Erlangen–Nürnberg Erlangen Germany

**Keywords:** ^23^Na RF birdcage coil, 7 Tesla, cardiac sodium (^23^Na) MRI, interleaved acquisition, parallel transmission (pTx)

## Abstract

**Purpose:**

To evaluate the feasibility of interleaved ^23^Na/^1^H cardiac MRI at 7 T using ^1^H parallel transmission (pTx) pulses.

**Methods:**

A combined setup consisting of a ^23^Na volume coil and two ^1^H transceiver arrays was employed and the transmit and receive characteristics were compared in vitro with those of the uncombined radiofrequency coils. Furthermore, the implemented interleaved ^23^Na/^1^H pTx sequence was validated in phantom measurements and applied to four healthy subjects. For the latter, three customized ^1^H excitation pulses (universal and individual phase shims (UPS/IPS) and individual 4kT pulses (4kT)) were employed in the interleaved ^23^Na/^1^H pTx sequence and compared with the vendor‐provided default cardiac phase shim (DPS).

**Results:**

Combining both coils resulted in a reduction of the mean ^23^Na transmit field (B_1_^+^) efficiency and ^23^Na signal‐to‐noise ratio by 18.9% and 15.4% for the combined setup, whereas the ^1^H B_1_^+^ efficiency was less influenced (−4.7%). Compared with single‐nuclear acquisitions, interleaved dual‐nuclear ^23^Na/^1^H MRI showed negligible influence on ^23^Na and ^1^H image quality. For all three customized ^1^H pTx pulses the B_1_^+^ homogeneity was improved (coefficients of variation: CV_UPS_ = 0.30, CV_IPS_ = 0.23, CV_4kT_ = 0.15) and no ^1^H signal dropouts occurred compared with the vendor‐provided default phase shim (CV_DPS_ = 0.37).

**Conclusion:**

The incorporation of customized ^1^H pTx pulses in an interleaved ^23^Na/^1^H sequence scheme was successfully demonstrated at 7 T and improvements of the ^1^H B_1_^+^ homogeneity within the heart were shown. Combining interleaved ^23^Na/^1^H MRI with ^1^H pTx is an important tool to enable robust quantification of myocardial tissue sodium concentrations at 7 T within clinically acceptable acquisition times.

## 1 INTRODUCTION

Sodium ions (Na^+^) play an essential role in many physiological processes and the Na^+^ concentration is closely linked to cell viability.^1^ For example, changes of the myocardial tissue sodium concentration (TSC) were observed in patients with myocardial infarction^2^ as well as primary hyperaldosteronism.^3^ Compared with interventional methods like myocardial biopsy, sodium (^23^Na) MRI currently represents the only technique to noninvasively determine the myocardial TSC in vivo.^4^

Compared to hydrogen (^1^H), ^23^Na shows significantly lower MR sensitivity and in vivo concentrations which, lead to a strongly reduced signal‐to‐noise ratio (SNR) for ^23^Na MRI (≈1:6000 in myocardium^4^). To compensate for this SNR deficit, larger voxel sizes and longer acquisition times are needed for ^23^Na MRI.^4^ In addition, ultrahigh field strengths are beneficial for ^23^Na MRI to further increase the SNR.^5^, ^6^

Due to the low spatial resolution of ^23^Na MRI, additional high‐resolution anatomical ^1^H MR data are required to enable a precise segmentation of different cardiac compartments (e.g., blood pool, myocardium) for partial volume correction^7^ and quantitative evaluation of the ^23^Na images.^8^ Previously, cardiac ^23^Na and ^1^H MRI were performed consecutively with different pulse sequences^4^, ^9^, ^10^ or even at different field strengths,^8^ which prolonged the total acquisition time. Additionally, the consecutive acquisition scheme is prone to changes in the physiological conditions of the subject (heart rate, respiratory amplitude and frequency) as well as prone to image coregistration errors.

Thus, in this work we used a dual‐nuclear interleaved ^23^Na/^1^H acquisition scheme^11^, ^12^ to receive both ^23^Na and ^1^H MR data within a single measurement at 7 T. For this, we used a combined coil setup consisting of a ^23^Na volume torso coil and two ^1^H torso arrays. This setup in combination with the interleaved ^23^Na/^1^H sequence allows to use the idle time of the ^23^Na acquisition to acquire additional anatomical ^1^H images. This reduces the total acquisition time, ensures alignment of the ^23^Na and ^1^H images without requiring image coregistration and guarantees that both images depict the quasi‐same physiological states. Simultaneous instead of interleaved ^23^Na/^1^H MR acquisition schemes, as demonstrated for ^23^Na/^1^H brain MRI,^13^ provide similar advantages, but would have required hardware modifications of the MR scanner used.

At 7 T, the excitation wavelength for ^1^H MRI is relatively short (≈11 cm in water^14^). This can lead to an inhomogeneous ^1^H excitation profile and even complete dropouts of the ^1^H signal within the heart,^15^ making a reliable segmentation difficult. To avoid this, we combined the interleaved ^23^Na/^1^H acquisition with the concept of multichannel parallel transmission (pTx)^16^, ^17^ for ^1^H excitation.

In the following, we investigated the influences of the combined coil setup and the interleaved ^23^Na/^1^H sequence. Subsequently, we applied the interleaved ^23^Na/^1^H sequence with different customized pTx pulses in four healthy subjects.

## 2 METHODS

### 2.1 Data acquisition and reconstruction

**FIGURE 1:**
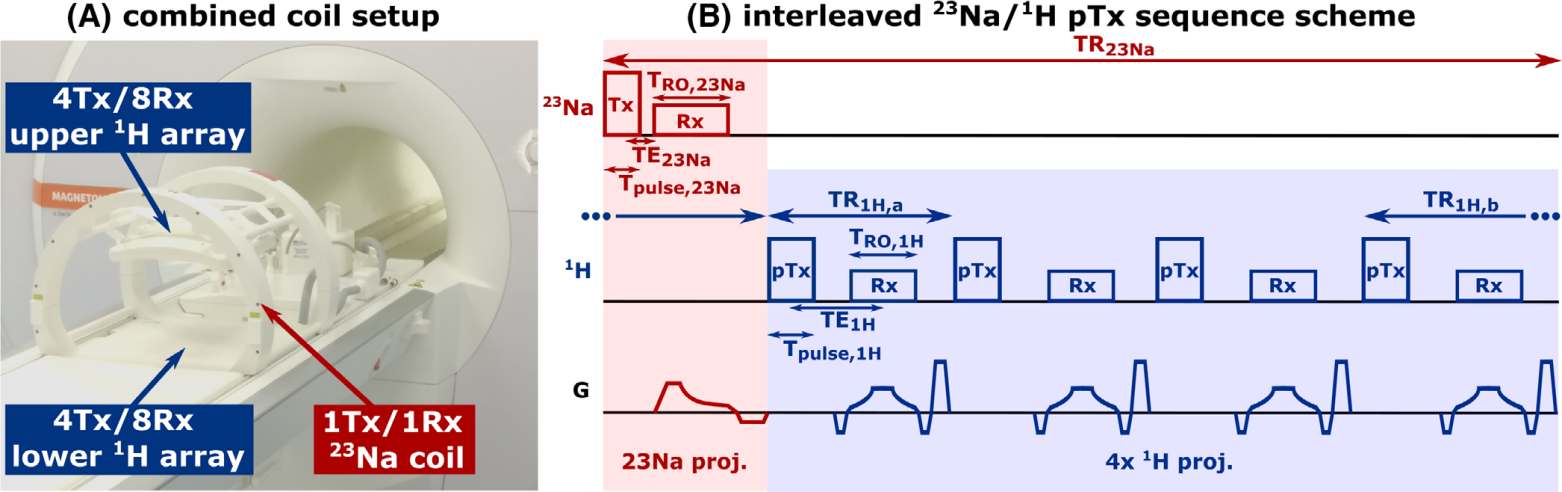
The combined radiofrequency coil setup (A) consists of a ^23^Na volume RF coil and two ^1^H arrays, each with four transmit (Tx) and eight receive (Rx) channels. The ^1^H arrays are positioned on top of the chest and below the back of the subject, and the multiple ^1^H Tx channels enable the use of parallel transmission (pTx) for the ^1^H excitation. In the interleaved ^23^Na/^1^H pTx sequence scheme (B), the acquisition of a ^23^Na projection is followed by the acquisition of four ^1^H projections during the idle time of the ^23^Na repetition time (TR). The excitation of ^23^Na is performed in 1Tx mode, while customized pTx pulses can be applied for the excitation of ^1^H.

All measurements were conducted on a 7T whole‐body system (Terra.X, Siemens Healthineers, Erlangen, Germany) using ^23^Na and ^1^H torso coils^18^ (Rapid Biomedical, Rimpar, Germany). For ^23^Na MRI, a volume coil was used, constructed as a four‐rung birdcage with asymmetric end rings adjusted to the magnet bore to provide maximum space for the subject.^18^ The ^1^H setup^15^ consists of two arrays, each with four transmit and eight receive channels, which are placed below the back and on top of the chest of the subject. Specific absorption rate (SAR) limits of the ^1^H arrays were met by limiting the time‐averaged radiofrequency (RF) power of each transmit channel to 1.195 W.^15^ For the ^23^Na coil, weight‐dependent (40≤50≤60≤80≤100 kg) SAR limits were used (1.05/2.1 W/kg times the minimum weight of each weight category for normal/first level operation mode). In addition, the component protection limits the maximum power of the ^23^Na coil to 120 W for the combined and 140 W for the uncombined setup. In addition to the individual use, both coils are also specifically designed to be operated in a combined setup (Figure 1A). Based on extensive testing of the coils (i.e., tuning, matching, and interactions between the coils at both the ^23^Na and ^1^H frequency), a declaration of safety and compatibility of the coil setup was provided by the manufacturer, including the setting of safe SAR limits for both the combined and uncombined coil setups.

For in vitro measurements, a torso‐like phantom (Na^+^ concentration ≈ 300 mM) was used mimicking the dielectric properties of the human torso.^19^ In vivo measurements were carried out on four healthy subjects (28 ± 3 years). All measurements were approved by the local ethics committee and each subject provided signed informed consent before the examination.

All MR data were acquired during free breathing and without cardiac triggering using a density‐adapted 3D radial readout^20^ and a golden angle^21^ projection scheme (Data S1). The reconstruction of all data was performed offline using a custom‐written MATLAB (The MathWorks, Natick, MA, USA) script. After application of a Hamming filter, all images were reconstructed using a nonuniform fast Fourier transform.^22^ In addition, the interleaved acquired ^23^Na images were interpolated to match the nominal spatial resolution of the interleaved acquired ^1^H images.

### 2.2 Interleaved ^23^Na/ ^1^H pTx sequence with customized ^1^H pTx pulses

In the dual‐nuclear interleaved ^23^Na/^1^H pTx sequence scheme (Figure 1B), one ^23^Na projection is followed by four ^1^H projections evenly distributed over the idle time of the ^23^Na repetition time.^12^ For ^23^Na MRI, we used a center‐out (half projection) radial readout enabling very short echo times to reduce signal loss resulting from the fast T_2_* decay of ^23^Na nuclei. Because T_2_* decay is slower for ^1^H, ^1^H projections were acquired in a center‐through (full projection) radial readout to ensure more efficient k‐space sampling. The corresponding sequence parameters of the interleaved ^23^Na/^1^H sequence can be found in Table 1. The B_0_ compensation of the scanner had to be turned off manually to avoid an image corruption of the interleaved acquired ^23^Na image (Figure S1).

**TABLE 1:** Acquisition parameters of the used MR sequences. In vitro absolute B_1_^+^ mapping was performed using the double‐angle (DA) method for ^23^Na and the actual flip angle imaging (AFI) method for ^1^H, respectively. In vivo relative channel‐wise three‐dimensional ^1^H B_1_^+^ maps were necessary for subject‐specific pTx pulse calculations (individual phase shim and individual 4kT‐points pulse). For the phase shims (default phase shim universal phase shim and individual phase shim), we used a ^1^H pulse duration of 2 ms, whereas for the individual 4kT‐points pulse the ^1^H pulse durations were 0.87 ms (Subjects 1–3) and 1.27 ms (Subject 4). For the interleaved ^23^Na/^1^H sequence, the excitation and readout parameters can be chosen independently for both nuclei. All in vivo measurements were performed during free breathing and without cardiac triggering.

	Interleaved ^23^Na/^1^H		^23^Na DA	^1^H AFI	Rel. ^1^H B_1_^+^
Application	In vitro/in vivo		In vitro		In vivo
Nucleus	^23^Na	^1^H	^23^Na	^1^H	^1^H
Readout scheme	DA‐3D‐RAD (half proj.)	DA‐3D‐RAD (full proj.)	DA‐3D‐RAD (half proj.)	DA‐3D‐RAD (full proj.)	DA‐3D‐RAD (full proj.)
Nominal spatial resolution [mm^3^]	6 × 6 × 6	2 × 2 × 2	12 × 12 × 12	4 × 4 × 4	4 × 4 × 4
Nominal FA [°]	82	10	45/90	70	8
Projections	15 000	60 000	4000	7500	10 000
TR [ms]	60	TR_1H,a_ = 13.08, TR_1H,b_ = 20.76	250	TR_1_ = 75, TR_2_ = 15	4.5
TE [ms]	1.15	2.5	1.15	3.03	2.02
T_pulse_ [ms]	2	DPS/UPS/IPS: 2, 4kT: 0.87/1.27	2	1	0.5
T_RO_ [ms]	5	2	5	2.5	2.5
Acquisition time (min:s)	15:00		2× 16:40	11:15	6:00

Abbreviations: AFI, actual flip angle imaging; DA, double angle method; DA‐3D‐RAD, density‐adapted 3D radial; FA, flip angle; TE, echo time; TR, repetition time; T_pulse_, transmit pulse duration; T_RO_, readout duration.

The employed interleaved sequence allows the application of pTx for the ^1^H excitation of the sequence. In this work, we used four different ^1^H excitation pulses^23^:

1. Default phase shim (DPS): vendor‐provided fixed phase shim for cardiac MRI
2. Universal phase shim (UPS): optimization of channel phases based on previously acquired cardiac B_1_^+^ maps of 35 volunteers^15^
3. Individual phase shim (IPS): optimization of channel phases based on additionally acquired B_1_^+^ maps of the measured subject^24^
4. Individual 4kT‐points pulse (4kT): optimization of amplitudes/phases of four sub‐pulses and intervening gradients based on additionally acquired B_1_^+^ maps of the measured subject^25^

The individual pulse optimization and the calculation of absolute flip angle (FA) maps were performed as described in Egger et al.^15^ The additional acquisition of B_1_^+^ maps and the computation of the individually optimized pTx pulses (IPS, 4kT) required about 10 min. A linear calibration fit based on 35 previously measured subjects (Figure S2) was employed for a subject‐specific estimation of the reference voltage based on the weight of the subject. The resulting reference voltage was used for all three phase shims.

### 2.3 B_1_ ^+^ and B_1_ ^−^ mapping methods

For the in vitro FA measurements of the ^23^Na volume coil, the double angle method^26^ was used (Table 1). The receive profile within the phantom was calculated based on the following formula^8^:

(1) $$B_{1}^{-}\left(\vec{x}\right)∼\frac{S\left(\vec{x}\right)}{\sin\left(\mathrm{FA}\left(\vec{x}\right)\right)}$$

with the measured signal S. For the ^1^H arrays, in vitro absolute FA maps were acquired using an actual flip angle imaging^27^ approach^15^ with the sequence parameters given in Table 1. The efficiency η of the transmit field (B_1_^+^) was calculated for both coils according to

(2) $$\eta \left(\vec{x}\right)=\frac{B_{1}^{+}\left(\vec{x}\right)}{U_{\text{applied}}}=\frac{\mathrm{FA}\left(\vec{x}\right)}{\gamma \cdot T_{\text{pulse}}\cdot U_{\text{applied}}}$$

with the applied pulse voltage $U_{\text{applied}}$. For the calculation of subject‐specific optimized ^1^H pTx pulses, additional relative channel‐wise three‐dimensional B_1_^+^ maps^28^ were acquired with a nominal spatial resolution of (4 mm)^3^ and an acquisition time of 6 min (see remaining sequence parameters in Table 1) as described by Egger et al.^15^ FA simulations were performed based on the measured channel‐wise three‐dimensional B_1_^+^ maps to calculate FA estimates for all four ^1^H excitation pulses.^15^ For better comparison of the homogeneity, the FA maps were normalized to the mean FA in the heart.^29^

### 2.4 Influence of combined setup

To quantify the mutual influence of the ^23^Na volume coil and the ^1^H arrays, ^23^Na and ^1^H B_1_^+^ efficiency maps and ^23^Na B_1_^−^ maps were measured in vitro for both the combined as well as uncombined coil setup. To assess the B_1_^+^ and B_1_^−^ homogeneity of the ^23^Na volume coil, the coefficient of variation (CV) was calculated within a central region of interest (132 × 228 × 204 mm^3^). Furthermore, voxelwise ^23^Na SNR maps were calculated based on the ^23^Na images, acquired with a nominal FA of 90°, and the standard deviation of additional noise scans within the phantom as follows^30^:

$$\mathrm{SNR}\left(\vec{x}\right)=\frac{{\text{image}}_{23\mathrm{Na}}\left(\vec{x}\right)}{{\mathrm{std}}_{\text{noise}}}.\sqrt{\frac{4-\pi }{2}}$$

To compare the performance of the combined to the uncombined setup, relative difference maps were calculated for the different coil properties X using the formula

$$\frac{\left(X_{\text{comb}}-X_{\text{uncomb}}\right)}{X_{\text{uncomb}}}$$

and the mean was computed over the whole phantom. External markers were used to ensure reproducible positioning of the phantom for both setups. In addition, the images of both setups were coregistered in the postprocessing using the Elastix toolbox.^31^

### 2.5 Influence of interleaved sequence

To investigate the influence of the interleaved ^23^Na/^1^H acquisition on the image quality of both nuclei, ^23^Na and ^1^H in vitro images acquired with the dual‐nuclear interleaved and single‐nuclear sequence were compared. For the single‐nuclear ^23^Na sequence, the ^1^H transmission and gradients in Figure 1B were switched off and vice versa for the single‐nuclear ^1^H sequence. Moreover, the mean ^23^Na SNR values were calculated for both sequences based on the corresponding images and additional noise scans as described above.^30^

## 3 RESULTS

### 3.1 Influence of the combined ^23^Na and ^1^H torso coil setup

**FIGURE 2:**
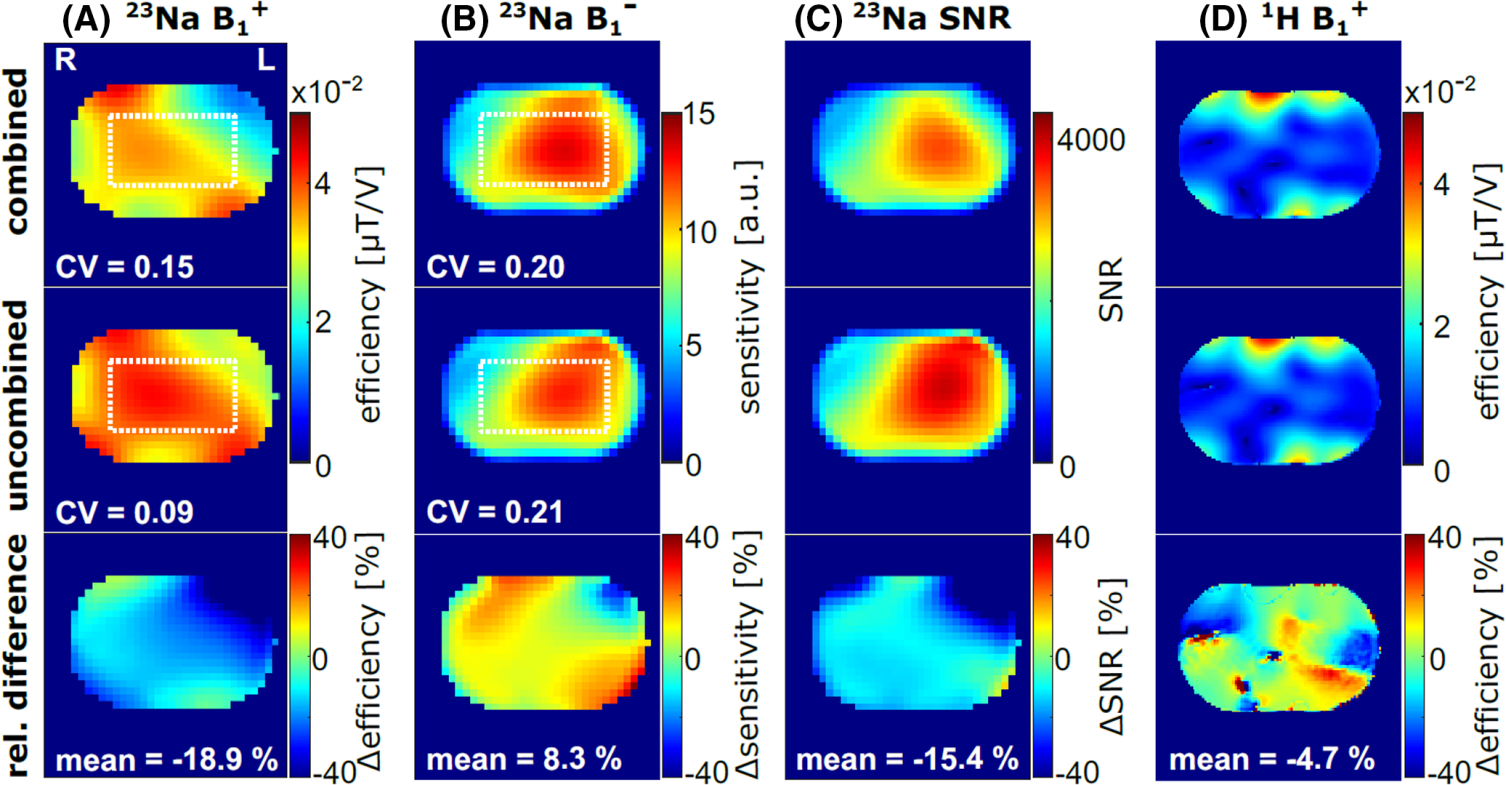
Comparison of the combined (first row) and uncombined (second row) application of the ^23^Na and ^1^H coil setups. To evaluate the B_1_^+^ and B_1_^−^ homogeneity of the ^23^Na volume coil, the coefficient of variation (CV) values were calculated within a central region of interest (volume of 132×228×204 mm^3^; white dotted box). Using the combined setup (CV_B1+, comb_ = 0.15), the ^23^Na B_1_^+^ (A) homogeneity was lower than for the uncombined case (CV_B1+, uncomb_ = 0.09), while homogeneity of the ^23^Na B_1_^−^ (B) distribution was less affected (CV _B1‐,comb_ = 0.20 vs CV _B1‐,uncomb_ = 0.21). The relative difference (third row) for each variable X was calculated by $\left(X_{\text{comb}}-X_{\text{uncomb}}\right)/X_{\text{uncomb}}$, and the mean difference was computed within the whole phantom. The mean ^23^Na B_1_^+^ efficiency (A) and ^23^Na SNR (C) were reduced by 18.9% and 15.4% for the combined setup, respectively. In contrast, ^1^H B_1_^+^ efficiency (D) was less influenced by the combination of both coils.

Figure 2 shows the mutual influence of the ^23^Na and ^1^H coils. Compared with the uncombined application of the ^23^Na volume coil, the mean ^23^Na B_1_^+^ efficiency was reduced by about 19% for the combined setup and especially dropped off toward the top left. Evaluation within a central region of interest displayed a higher CV and thus a decrease in ^23^Na B_1_^+^ homogeneity for the combined setup (CV = 0.15 vs. 0.09), whereas ^23^Na B_1_^−^ homogeneity remained similar for both setups (CV = 0.20 vs. 0.21). For the combined setup, the ^23^Na noise level was elevated by 24% compared with the uncombined coil setup, such that the ^23^Na SNR showed a mean reduction of 15.4%. The additional ^23^Na body coil had only a minor effect on the ^1^H B_1_^+^ efficiency (−4.7%) and showed no relevant influence on customized ^1^H pTx pulses (Figure S3).

### 3.2 Influence of the dual‐nuclear interleaved ^23^Na/ ^1^H acquisition

**FIGURE 3:**
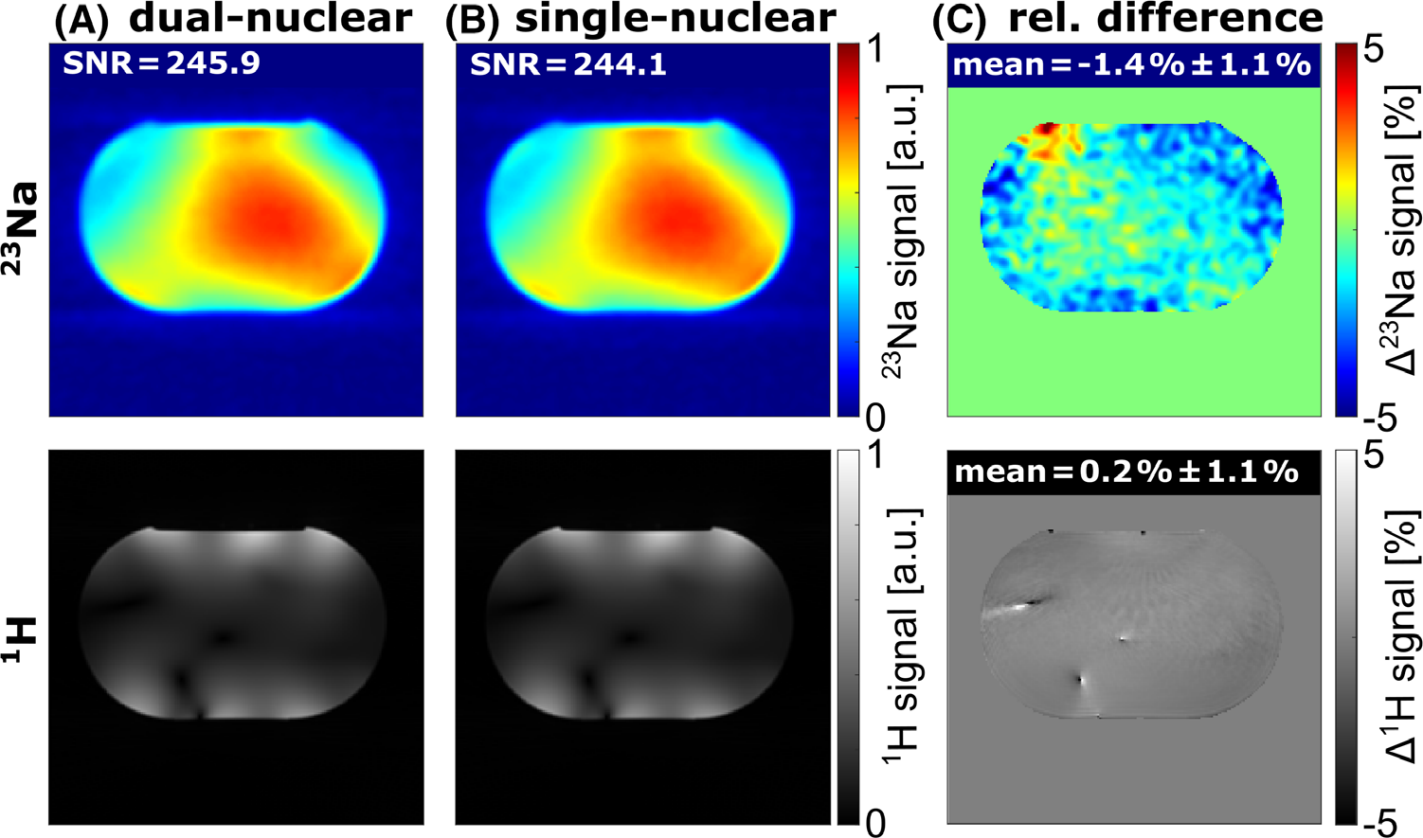
Comparison of ^23^Na and ^1^H images acquired with the dual‐nuclear ^23^Na/^1^H interleaved (A) and single‐nuclear sequences (B) using the combined ^23^Na/^1^H coil setup. For the single‐nuclear ^23^Na sequence, the excitation and gradients for ^1^H were turned off in the interleaved sequence and vice versa for the single‐nuclear ^1^H sequence. For ^1^H excitation, the vendor‐provided default phase shim was used. (C) The relative signal difference was obtained by $\left(S_{\text{dual}}-S_{\text{single}}\right)/S_{\text{single}}$, and the mean relative signal difference was calculated over the whole phantom. For both nuclei, no relevant differences were visible.

Figure 3 depicts the influence of the dual‐nuclear interleaved ^23^Na/^1^H acquisition on both images compared with the corresponding single‐nuclear acquisitions. For both ^23^Na and ^1^H images, no relevant differences were found. The mean relative signal difference over the whole phantom was −1.4%±1.1% for ^23^Na and 0.2%±1.1% for ^1^H. The SNR of the ^23^Na image was not affected by the additional acquisition of ^1^H MR data in the dual‐nuclear interleaved sequence.

### 3.3 In vivo application of the interleaved ^23^Na/ ^1^H pTx sequence

**FIGURE 4:**
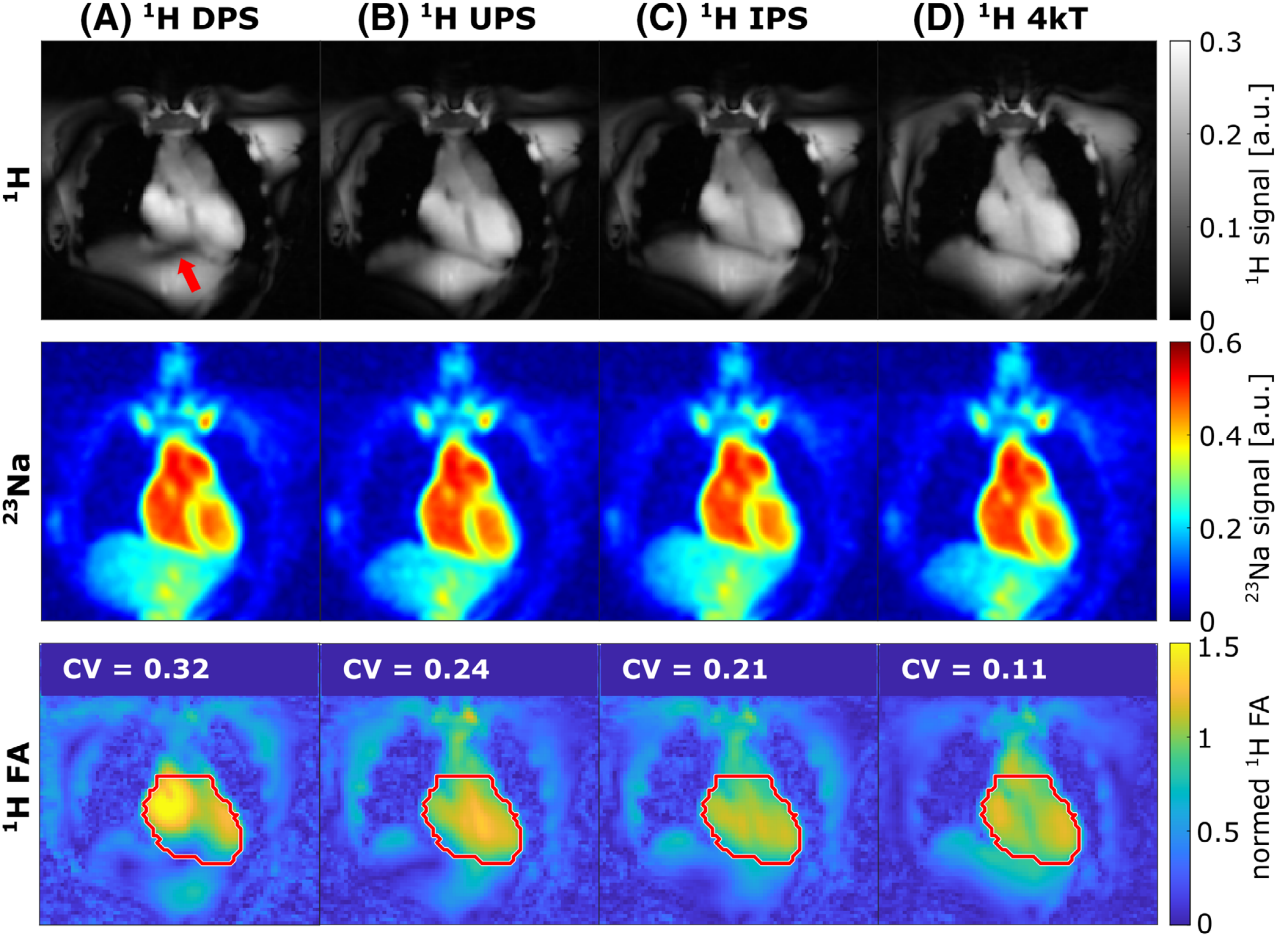
In vivo interleaved ^23^Na/^1^H pTx MR images (acquisition time of 15 min) of one healthy subject for different ^1^H excitation pulses during free breathing and without cardiac triggering. While for the vendor‐provided DPS (A) ^1^H signal dropouts (red arrow) were visible within the heart, all three customized pTx pulses (B–D) showed no ^1^H signal dropouts (first row). The improvements in ^1^H flip angle (FA) homogeneity are also demonstrated by the reduced coefficient of variation (CV) values calculated within the heart volume (red line) of the corresponding simulated ^1^H FA maps (third row). For better comparison of the homogeneity, the simulated ^1^H FA maps were normalized to the mean ^1^H FA within the heart. ^23^Na images were unaffected by the choice of the ^1^H excitation pulse (second row). DPS, default phase shim; IPS, individual phase shim; UPS, universal phase shim; 4kT, 4kT‐points pulse.

Figure 4 shows an exemplary in vivo application of the interleaved ^23^Na/^1^H pTx sequence for one subject. For the acquisition with the vendor‐provided DPS signal dropouts (red arrow) in the heart were visible in the ^1^H image. For all three customized pTx pulses (UPS, IPS and 4kT), no ^1^H signal dropouts occurred (first row), which is also shown in the simulated ^1^H FA map (third row). Averaged over all four measured subjects (Table S1), the UPS showed enhanced homogeneity with reduced CV (CV = 0.30±0.05) compared with the DPS (CV = 0.37±0.08). The IPS yielded further improvements (CV = 0.23±0.02) and the best homogeneity was achieved with the 4kT pulses (CV = 0.15±0.03). The animated overlay of the ^23^Na and ^1^H images in Figure S4 demonstrates that both images are aligned without the need for image coregistration.

## 4 DISCUSSION

In this work, we demonstrated the feasibility of interleaved ^23^Na/^1^H pTx cardiac MRI at 7 T. Apart from interleaving ^1^H MRI with X‐nuclei MR spectroscopy,^11^, ^32^ interleaved X‐nuclei/^1^H MRI in general has only been applied to a few different regions of the human body^11^ such as knee,^33^, ^34^ lung,^35^, ^36^ breast^37^ and brain.^12^, ^29^, ^38^ However, an interleaved X‐nuclei/^1^H MRI acquisition is especially beneficial for cardiac MRI. The latter is challenged by image artifacts caused by respiratory and cardiac motion. Thus, physiological changes, such as from shallow to heavy breathing, between consecutive ^23^Na and ^1^H cardiac measurements may falsify the TSC quantification. Furthermore, for ^23^Na and ^1^H cardiac MRI at different field strengths,^8^ errors in the image coregistration, which has to be performed to correct for the different positioning of the subject, might also affect the TSC quantification. Using an interleaved ^23^Na/^1^H acquisition scheme, these described error sources can be avoided, as ^23^Na and ^1^H images are acquired within one measurement. Another main advantage of the interleaved acquisition is the reduced total scan time, which can be particularly important for patient studies. In summary, interleaved ^23^Na/^1^H cardiac MRI will be an improvement toward a fast and precise quantification of the myocardial TSC.

However, cardiac ^1^H MRI at 7 T is challenging due to the B_1_^+^ inhomogeneity at the high field strength.^39^ So far, interleaved X‐nuclei/^1^H MRI has only been performed with 1Tx and capabilities of pTx were not exploited.^11^ Thus, we incorporated, to our knowledge for the first time, the concept of ^1^H pTx in an interleaved ^23^Na/^1^H sequence at 7 T and included different customized ^1^H pTx pulses. The implemented interleaved sequence allows for the application of individually or universally optimized ^1^H phase shims as well as more sophisticated dynamic kT‐points pTx pulses, additionally optimizing the pulse amplitudes and/or gradients between subpulses, depending on the target application. For fast applications, a UPS or even a universal kT‐points pulse^25^ would be advisable. If additional ^1^H B_1_^+^ mapping, which could be accelerated^15^, ^40^ in the future, and pulse calculation are feasible in terms of time, one could use individually optimized pTx pulses. As confirmed in this work, these usually outperform universal pulses.^25^, ^41^ Compared with the vendor‐provided DPS, no ^1^H signal dropouts occurred for all three customized ^1^H pTx pulses used in this work. This ensures a precise segmentation of different cardiac compartments in the ^1^H images, which is necessary for a reliable quantification of the myocardial TSC. Furthermore, not being limited to a vendor‐provided fixed cardiac phase shim facilitates the transfer of the interleaved ^23^Na/^1^H acquisition to other abdominal applications by using customized ^1^H pTx pulses optimized for the specific target region.

Compared with the uncombined application of the ^23^Na volume coil, for the combined setup the B_1_^+^ efficiency was reduced by an average of 19%, whereas the B_1_^−^ sensitivity increased by about 8%. Even the uncombined ^23^Na coil showed different B_1_^+^ and B_1_^−^ field distributions, probably due to an asymmetry between the horizontal and vertical (linear) modes of the volume coil, which may be caused, for example by the different proximity to the bore. The additional ^1^H arrays in the combined setup might further increase this asymmetry, leading to the observed deviations of the B_1_^+^ and B_1_^−^ field distributions compared with the uncombined setup. The SNR reduction of 15% for the combined coil setup arises primarily from a higher noise level for the combined setup. In contrast to the ^23^Na coil, the B_1_^+^ efficiency of the ^1^H arrays was less influenced by the combination of both coils, as the very large ^23^Na coil appears to act more like a ground plane for the ^1^H arrays. In the work of Lott et al.,^8^ the ^23^Na B_1_^+^ and B_1_^−^ correction had only a minor effect on the quantification of the myocardial TSC. However, for our combined setup (CV_B1+_ = 0.15, CV_B1‐_ = 0.20), the ^23^Na B_1_^+^ and B_1_^−^ variations may have a greater impact on quantification than for their coil setup (CV_B1+_ = 0.07, CV_B1‐_ = 0.08^19^). Thus, additional acquisition of ^23^Na B_1_^+^ maps might be necessary to correct for B_1_^+^ variations in quantitative TSC evaluations.^8^ However, this would not be possible for B_1_^−^ maps, as these can only be acquired in a sample with homogeneous sodium concentration. In the future, a B_1_^−^ correction based on electromagnetic field simulations^8^ could be promising. Furthermore, an additional dielectric pad^42^ might reduce the drop‐off of the ^23^Na B_1_^+^ and B_1_^−^ field in the upper left of the phantom. Regarding the coil setup, the used ^23^Na volume coil should provide a more homogeneous B_1_^+^ and B_1_^−^ field compared with multichannel ^23^Na arrays. In contrast, the latter would result in an increased ^23^Na SNR as demonstrated by Kaggie et al.^43^ Thus, our combined coil setup offers the option to incorporate an additional receive‐only ^23^Na array in the future, which could potentially be integrated directly in the anterior ^1^H array. Apart from that, dual‐tuned ^23^Na/^1^H transceiver arrays might be an interesting alternative.^44^

Despite the additional gradients of the dual‐nuclear sequence, no relevant differences were observed in neither the ^23^Na nor ^1^H images compared with the images acquired with the single‐nuclear sequences, which agrees with previous publications.^12^, ^33^ The SNR of the ^23^Na images was also not affected by the interleaved acquisition. This is in accordance with previously published results of a similar interleaved ^23^Na/^1^H sequence scheme acquired with a dual‐tuned ^23^Na/^1^H head coil.^12^ The Terra.X scanner version enables interleaved ^23^Na/^1^H MRI with customized ^1^H pTx pulses, unlike previous software versions, which only supported interleaved ^23^Na/^1^H MRI with ^1^H excitation in 1Tx mode. Apart from general X‐nuclei modifications like a broadband amplifier and dedicated coils, the interleaved ^23^Na/^1^H pTx sequence was applied without any additional hardware modifications that were required in previous studies for interleaved or simultaneous acquisitions.^13^, ^45^ However, due to software incompatibilities, similar to the incompatibility of the eddy current compensation for X‐nuclei described in McLean et al.,^46^ the vendor‐provided B_0_ compensation had to be turned off manually, but this should be fixed with the next software update.

One limitation of dual‐nuclear interleaved ^23^Na/^1^H acquisitions is the increased SAR due to the additional ^1^H excitation pulses compared with single‐nuclear ^23^Na acquisitions. This can be considered in the optimization of the ^23^Na pulse sequence parameters.^47^ In addition, more sophisticated SAR models such as virtual observation points^48^ might be used in the future. When considering local SAR, the interleaved ^23^Na/^1^H sequence can take advantage of the different spatial distributions of the SAR hotspots for each nucleus. The SAR would be determined by the maximum of the combined local SAR distribution, potentially resulting in a lower SAR than the cumulated SAR of both single‐nuclear sequences.^49^

Another challenge in cardiac ^23^Na/^1^H MRI are artifacts caused by respiratory and cardiac motion. The acquisition of all data in free breathing and without cardiac triggering leads to errors in the quantification of the myocardial TSC.^8^ As a potential solution, additional data points in the k‐space center could be acquired for each ^23^Na/^1^H projection. This may allow to apply retrospective self‐gating methods^19^ to reconstruct respiratory and cardiac sorted ^23^Na/^1^H images. Due to the interleaved acquisition scheme, the ^1^H images might then be used for motion correction of the ^23^Na data^12^ in the future.

## 5 CONCLUSION

In this work, we successfully demonstrated the application of an interleaved ^23^Na/^1^H MR acquisition with customized ^1^H pTx pulses for cardiac MRI at 7 T. Using customized ^1^H pTx pulses in the presented interleaved ^23^Na/^1^H pTx sequence enabled the time‐efficient acquisition of cardiac ^23^Na and ^1^H images within one measurement, while ensuring homogeneous ^1^H excitation of the heart.

## CONFLICT OF INTEREST

One of the co‐authors (Titus Lanz) is an employee of Rapid Biomedical GmbH.

## Supporting information


**Data S1.** Formulas to calculate the polar $\phi_{n}$ and azimutal angles $\theta_{n}$ and Cartesian unit vectors of the radial projections (n = 1, …, 15 000 for ^23^Na; and n = 1, …, 60 000 for ^1^H) based on the two‐dimensional (2D) golden means ($\lambda_{1}=0.4656$, $\lambda_{2}=0.6823$) presented by Chan et al.^21^
**Figure S1.** Influence of vendor‐provided B_0_ compensation on interleaved ^23^Na/^1^H MRI. In (A) and (B), measurements were performed under the B_0_ compensation currently provided by the vendor, whereas (C) was performed with a corrected software implementation of the B_0_ compensation at another Terra.X development device of the vendor. All measurements were performed using a ^23^Na resolution phantom and a dual‐tuned ^23^Na/^1^H head coil. The same interleaved sequence parameters as for the in vitro and in vivo cardiac measurements were used for (A) and (B), whereas for (C) the resolution of the ^23^Na images was (4 mm)^3^ instead of (6 mm)^3^.
(A) For ^23^Na MRI, we used a single‐nuclear ^23^Na sequence (I) only containing transmission, readouts, and gradients for ^23^Na as reference image. Acquiring a ^23^Na image using the dual‐nuclear interleaved ^23^Na/^1^H sequence with turned on B_0_ compensation (II) leads to a spatial shifting and blurring of the ^23^Na image (II‐I). By manually turning off the B_0_ compensation (III), these effects can be avoided, and there are no relevant differences between the ^23^Na image of the single‐nuclear and dual‐nuclear sequence (III‐I; mean difference over the phantom: 0.62% of the maximum value).
(B) For ^1^H MRI, B_0_ compensation worked for single‐nuclear and dual‐nuclear sequences (not shown here). Therefore, we used the interleaved acquired ^1^H image as reference (I). Because we had to turn off the B_0_ compensation for interleaved ^23^Na/^1^H acquisitions due to the artifacts for the ^23^Na image, interleaved acquired ^1^H images were not B_0_ compensated (II), resulting in minor spatial shifts visible in the difference image (II‐I). However, using image coregistration (III), these shifts could be corrected (III‐I; mean difference over the phantom: −0.02% of the maximum value). Because the effect of the B_0_ compensation is only determined by the shapes and timings of the applied gradients, it can be assumed that these shifts are constant for repeated measurements with the same gradient scheme. Even though the shifts were in the submillimeter range (Δx=−0.22mm,Δy=0.13mm,Δz=0.35mm), and thus almost negligible, we corrected the interleaved acquired ^1^H images for these shifts in our reconstruction.
After reporting the problem to the vendor, they identified errors in the software implementation of the B_0_ compensation that affect X‐nuclei MRI during dual‐nuclear interleaved acquisitions. (C) Test measurements of the interleaved sequence acquired under the latest software version with a corrected software implementation of the B_0_ compensation on another Terra.X development device of the vendor. There were no differences (II‐I) visible between single (I) and dual‐nuclear (II) measurements acquired with turned on *B*_*0*_ compensation. As for (A), the differences between the single‐nuclear sequence with turned‐on B_0_ compensation and the dual‐nuclear interleaved sequence with turned‐off B_0_ compensation did not show relevant differences (III‐I). In summary, after the next software update of our scanner, the interleaved sequence should be applicable without turning off the B_0_ compensation. However, until then, we have to use the presented work‐around of manually turning off the B_0_ compensation for the interleaved measurements.
**Figure S2.** Linear calibration fit ($U_{\mathrm{ref}}=9.4\;V/\mathrm{kg}\cdot \text{weight}+542.5\;V$) between the calculated ^1^H reference voltages within the heart for the default phase shim (DPS) and the body weight of 35 previously measured subjects.^15^ The linear fit showed good correlation between the reference voltage within the heart and the weight of the subjects (correlation factor: 0.76). The relative absolute deviation between the measured reference voltage and the corresponding reference voltage based on the calibration fit was calculated by $\frac{\left|U_{\mathrm{fit}}-U_{\text{meas}}\right|}{U_{\text{meas}}}$. For the 35 subjects, the mean relative absolute deviation was 6.4% (minimal/maximal: 0.02%/14.5%). Because there are currently no inline adjustments available for ^1^H body imaging at 7 T, we used this calibration fit to estimate subject‐specific ^1^H reference voltages for the DPS, universal phase shim (UPS), and individual phase shim (IPS) based on the weight of the measured subjects. However, due to the increased B_1_^+^ efficiency of UPS and IPS,^15^ an additional calibration fit for the UPS and direct calculation of the reference voltage during the optimization of the IPS could further improve the estimation of the flip angle (FA) in the future.
**Figure S3.** (A) Comparison of measured in vitro channel‐wise relative ^1^H B_1_^+^ maps for the combined and uncombined coil setup. The channel‐wise relative ^1^H B_1_^+^ maps of the combined setup were image‐coregistered to the uncombined setup, reducing misalignments of the phantom due to the repositioning. Absolute difference maps were calculated by $B_{1,\text{comb}}^{+}-B_{1,\text{uncomb}}^{+}$, and the mean difference was calculated within an exemplary heart region (red line, Subject 1). Within the heart region, no relevant differences were visible. (B) Influence of coil setups on optimized ^1^H excitation pulses. Using the shown in vitro channel‐wise relative ^1^H B_1_^+^ maps in (A), ^1^H phase shims were optimized for each coil setup (combined, uncombined) within the same three‐dimensional (3D) heart region (red line, Subject 1) and then applied to the channel‐wise relative ^1^H B_1_^+^ maps of both setups. For individually designed pulses, the pulses are usually optimized during the measurement and thus applied on the same coil setup. Coefficient of variation (CV) values showed no relevant differences between ^1^H pulses designed and applied on the uncombined (I) and combined setup (IV). Even pulses optimized on relative B_1_^+^ maps of one of the coil setups and then applied to the other coil setup (II, III) yielded comparable CV values. Thus, for the performance of ^1^H pulses optimized within the heart region, the influence of the ^23^Na coil in the combined setup appears to be negligible.
**Figure S4.** Animated overlay of ^23^Na and ^1^H images (Subject 1) acquired with the interleaved ^23^Na/^1^H pTx sequence using the universal phase shims (UPS) for ^1^H excitation. Due to the interleaved ^23^Na/^1^H acquisition scheme, the corresponding ^23^Na and ^1^H images are aligned without the need for image coregistration.
**Table S1.** Overview of coefficient of variation (CV) values. The mean CV values were calculated within the heart volume based on the simulated ^1^H FA maps of each subject and are shown for the four different ^1^H excitation pulses (default phase shim (DPS), universal phase shim (UPS), individual phase shim (IPS) and individual 4kT pulses (4kT)) of each subject.
