# Supplementary material for: Interleaved 23 Na/ 1H MRI of the human heart at 7 T using a combined 23Na/ 1H coil setup and 1H parallel transmission
Source: Magn Reson Med. 2025 Mar 10;94(1):231–41. doi: 10.1002/mrm.30426 (PMC12021321; doi:10.1002/mrm.30426)
Supplement: Supplementary file 1 — Data S1. Formulas to calculate the polar ϕn and azimutal angles θn and Cartesian unit vectors of the radial projections (n = 1, …, 15 000 for 23Na; and n = 1, …, 60 000 for 1H) based on the two‐dimensional (2D) golden means (λ1=0.4656, λ2=0.6823) presented by Chan et al. 21 Figure S1. Influence of vendor‐provided B0 compensation on interleaved 23Na/1H MRI. In (A) and (B), measurements were performed under the B0 compensation currently provided by the vendor, whereas (C) was performed with a corrected software implementation of the B0 compensation at another Terra.X development device of the vendor. All measurements were performed using a 23Na resolution phantom and a dual‐tuned 23Na/1H head coil. The same interleaved sequence parameters as for the in vitro and in vivo cardiac measurements were used for (A) and (B), whereas for (C) the resolution of the 23Na images was (4 mm)3 instead of (6 mm)3. (A) For 23Na MRI, we used a single‐nuclear 23Na sequence (I) only containing transmission, readouts, and gradients for 23Na as reference image. Acquiring a 23Na image using the dual‐nuclear interleaved 23Na/1H sequence with turned on B0 compensation (II) leads to a spatial shifting and blurring of the 23Na image (II‐I). By manually turning off the B0 compensation (III), these effects can be avoided, and there are no relevant differences between the 23Na image of the single‐nuclear and dual‐nuclear sequence (III‐I; mean difference over the phantom: 0.62% of the maximum value). (B) For 1H MRI, B0 compensation worked for single‐nuclear and dual‐nuclear sequences (not shown here). Therefore, we used the interleaved acquired 1H image as reference (I). Because we had to turn off the B0 compensation for interleaved 23Na/1H acquisitions due to the artifacts for the 23Na image, interleaved acquired 1H images were not B0 compensated (II), resulting in minor spatial shifts visible in the difference image (II‐I). However, using image coregistration (III), these shifts could be corrected [file MRM-94-231-s001.docx]

**Supporting Information**

$$\phi_{n}=\arccos\left( -1+modulo\left( 2\cdot\left( n-1 \right)\cdot\lambda_{1},2 \right) \right)$$

$$\theta_{n}=modulo(\left( n-1 \right)\cdot\lambda_{2}\cdot2\pi, 2\pi)$$

$$e_{x,n}=\sin\left( \phi_{n} \right)\cdot sin \left( \theta_{n} \right)$$

$$e_{y,n}=\sin\left( \phi_{n} \right)\cdot cos \left( \theta_{n} \right)$$

$$e_{z,n}=\cos\left( \phi_{n} \right)$$

*Information S1: Formulas to calculate the polar* $\phi_{n}$ *and azimutal angles* $\theta_{n}$ *and Cartesian unit vectors of the radial projections (n =1,…,15000 for ^23^Na and n=1,…,60 000 for ^1^H) based on the 2D golden means (*$\lambda_{1}=0.4656$*,* $\lambda_{2}=0.6823$*) presented by Chan et al.^21^.*


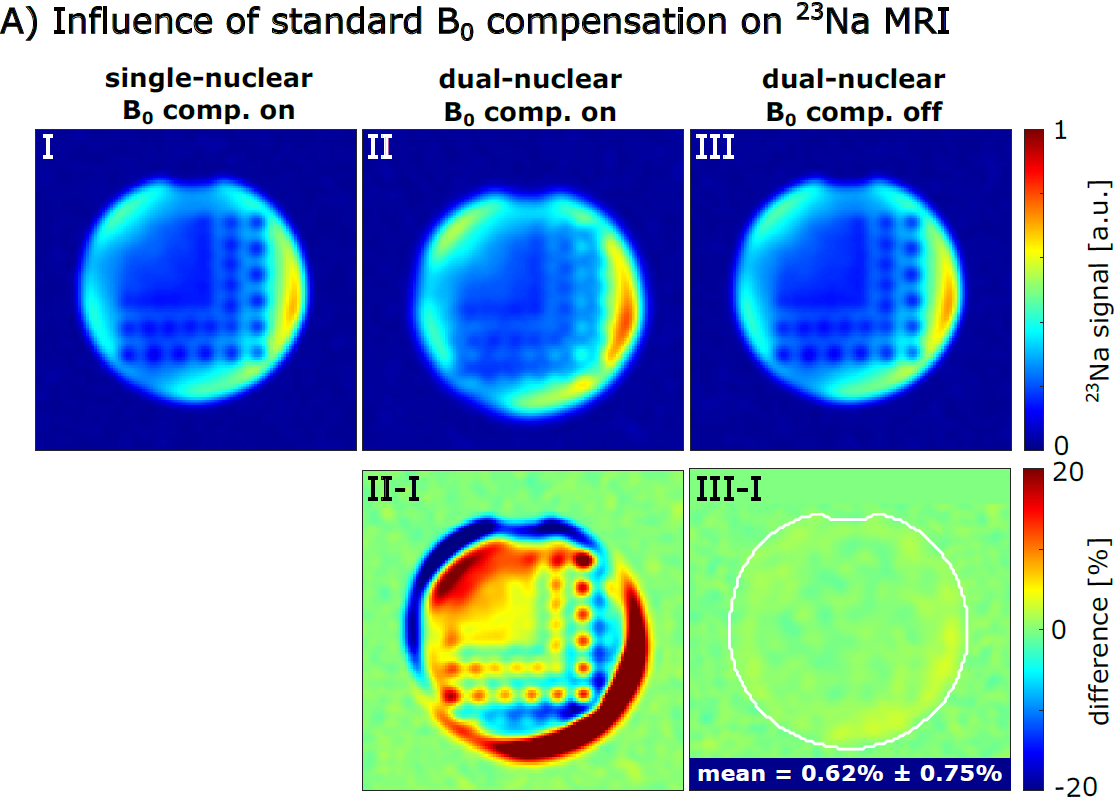

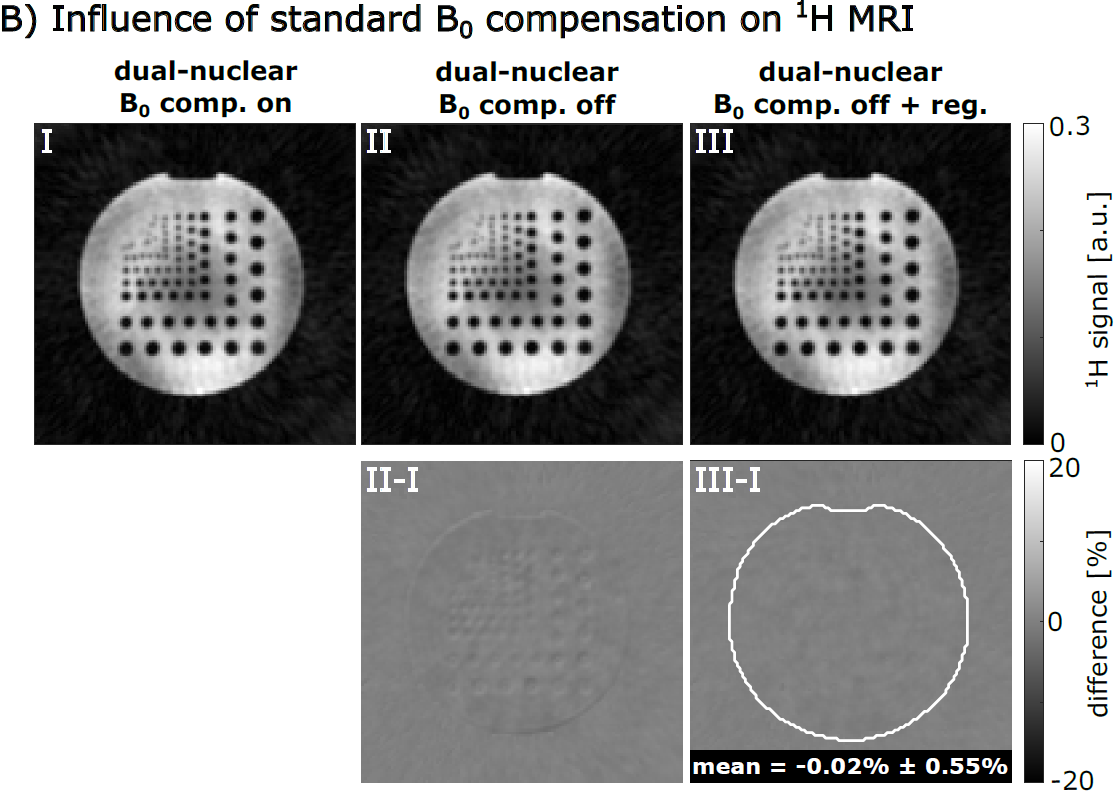

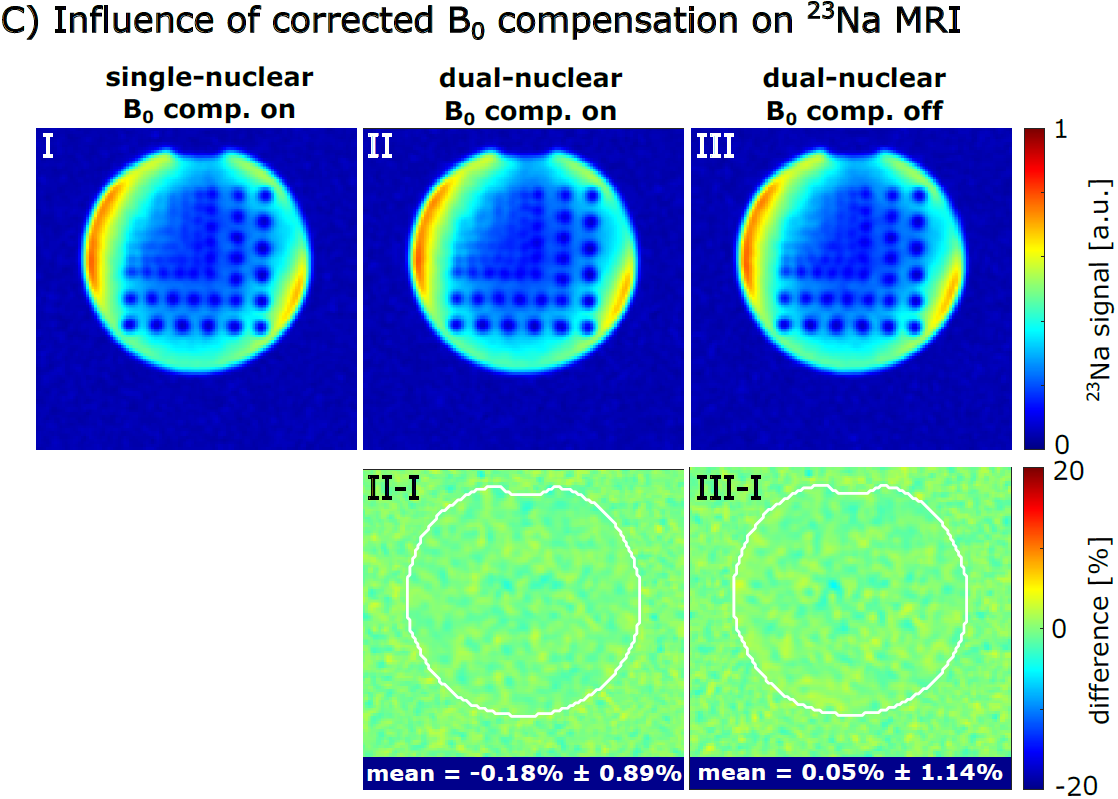


Figure S1: Influence of vendor-provided B_0_ compensation on interleaved ^23^Na/^1^H MRI. In A) and B) measurements were performed under the B_0_ compensation currently provided by the vendor, while C) was performed with a corrected software implementation of the B_0_ compensation at another Terra.X development device of the vendor. All measurements were performed using a ^23^Na resolution phantom and a dual-tuned ^23^Na/^1^H head coil. The same interleaved sequence parameters as for the in vitro and in vivo cardiac measurements were used for A) and B), while for C) the resolution of the ^23^Na images was (4 mm)^3^ instead of (6 mm)^3^.

(A) For ^23^Na MRI we used a single-nuclear ^23^Na sequence (I) only containing transmission, readouts and gradients for ^23^Na as reference image. Acquiring a ^23^Na image using the dual-nuclear interleaved ^23^Na/^1^H sequence with turned on B_0_ compensation (II) leads to a spatial shifting and blurring of the ^23^Na image (II-I). By manually turning off the B_0_ compensation (III), these effects can be avoided and there are no relevant differences between the ^23^Na image of the single-nuclear and dual-nuclear sequence (III-I, mean difference over the phantom: 0.62% of the maximum value).

(B) For ^1^H MRI, B_0_ compensation worked for single-nuclear and dual-nuclear sequences (not shown here). Therefore, we used the interleaved acquired ^1^H image as reference (I). Since we had to turn off the B_0_ compensation for interleaved ^23^Na/^1^H acquisitions due to the artifacts for the ^23^Na image, interleaved acquired ^1^H images were not B_0_ compensated (II), resulting in minor spatial shifts visible in the difference image (II-I). However, using image co-registration (III), these shifts could be corrected (III-I, mean difference over the phantom: -0.02% of the maximum value). Since the effect of the B_0_ compensation is only determined by the shapes and timings of the applied gradients, it can be assumed that these shifts are constant for repeated measurements with the same gradient scheme. Even though the shifts were in the submillimeter range ($\Delta x=-0.22 mm, \Delta y=0.13 mm, \Delta z=0.35 mm$) and thus almost negligible, we corrected the interleaved acquired ^1^H images for these shifts in our reconstruction.

After reporting the problem to the vendor, they identified errors in the software implementation of the B_0_ compensation that affect X-nuclei MRI during dual-nuclear interleaved acquisitions. (C) Test measurements of the interleaved sequence acquired under the latest software version with a corrected software implementation of the B_0_ compensation on another Terra.X development device of the vendor. There were no differences (II-I) visible between single (I) and dual-nuclear (II) measurements acquired with turned on B_0_ compensation. As for A), the differences between the single-nuclear sequence with turned on B_0_ compensation and the dual-nuclear interleaved sequence with turned off B_0_ compensation did not show relevant differences (III-I). In summary, after the next software update of our scanner the interleaved sequence should be applicable without turning off the B_0_ compensation. However, until then we have to use the presented work-around of manually turning off the B_0_ compensation for the interleaved measurements.


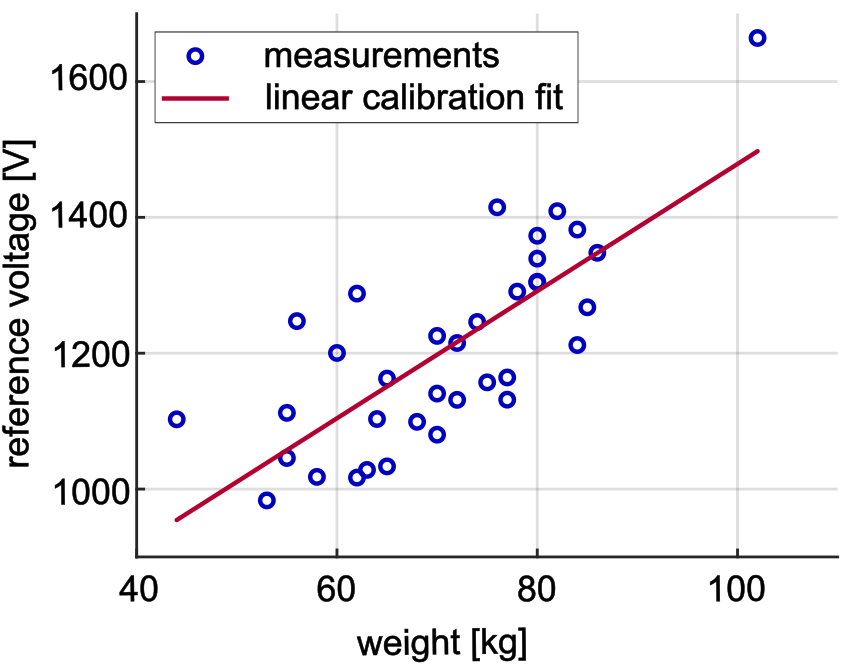


Figure S2: Linear calibration fit ($U_{ref}=9.4 V/kg\cdot weight+542.5 V$) between the calculated ^1^H reference voltages within the heart for the DPS and the body weight of 35 previously measured subjects^15^. The linear fit showed good correlation between the reference voltage within the heart and the weight of the subjects (correlation factor: 0.76). The relative absolute deviation between the measured reference voltage and the corresponding reference voltage based on the calibration fit was calculated by $\frac{\left| U_{fit}-U_{meas} \right|}{U_{meas}}$. For the 35 subjects the mean relative absolute deviation was 6.4% (minimal/maximal: 0.02% / 14.5%). Since there are currently no inline adjustments available for ^1^H body imaging at 7 T, we used this calibration fit to estimate subject-specific ^1^H reference voltages for the DPS, UPS and IPS based on the weight of the measured subjects. However, due to the increased B_1_^+^ efficiency of UPS and IPS^15^ an additional calibration fit for the UPS and direct calculation of the reference voltage during the optimization of the IPS could further improve the estimation of the FA in the future.


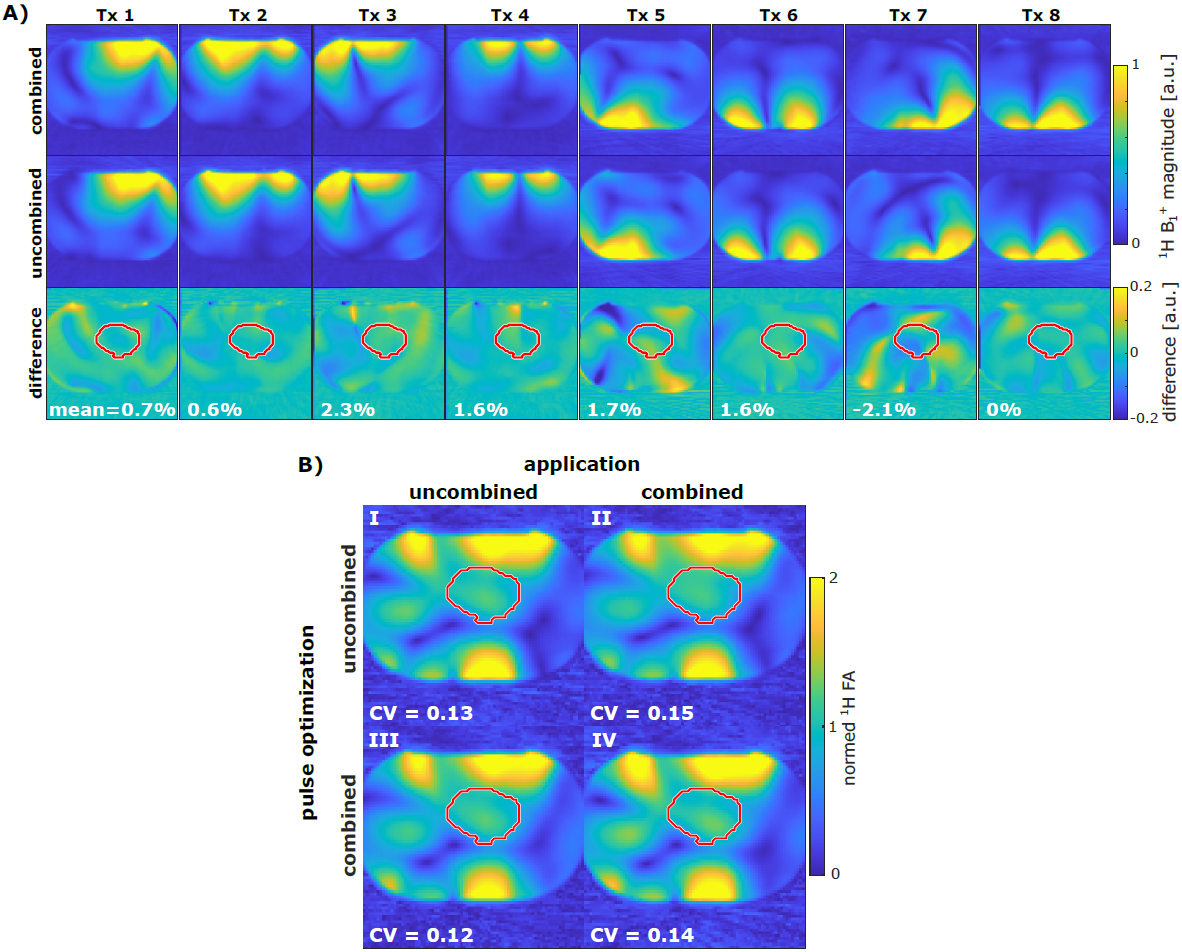


Figure S3: (A) Comparison of measured in vitro channel-wise relative ^1^H B_1_^+^ maps for the combined and uncombined coil setup. The channel-wise relative ^1^H B_1_^+^ maps of the combined setup were image co-registered to the uncombined setup, reducing misalignments of the phantom due to the repositioning. Absolute difference maps were calculated by $B_{1,comb}^{+}-B_{1,uncomb}^{+}$ and the mean difference was calculated within an exemplary heart region (red line, subject 1). Within the heart region no relevant differences were visible.

(B) Influence of coil setups on optimized ^1^H excitation pulses. Using the shown in vitro channel-wise relative ^1^H B_1_^+^ maps in (A), ^1^H phase shims were optimized for each coil setup (combined, uncombined) within the same 3D heart region (red line, subject 1) and then applied to the channel-wise relative ^1^H B_1_^+^ maps of both setups. For individually designed pulses, the pulses are usually optimized during the measurement and thus applied on the same coil setup. CV values showed no relevant differences between ^1^H pulses designed and applied on the uncombined (I) and combined setup (IV). Even pulses, optimized on relative B_1_^+^ maps of one of the coil setups and then applied to the other coil setup (II,III), yielded comparable CV values. Thus, for the performance of ^1^H pulses, optimized within the heart region, the influence of the ^23^Na coil in the combined setup appears to be negligible.

Table S1: Overview of CV values. The mean CV values were calculated within the heart volume based on the simulated ^1^H FA maps of each subject and are shown for the four different ^1^H excitation pulses (default phase shim (DPS), universal phase shim (UPS), individual phase shim (IPS) and individual 4kT-points pulse (4kT)) of each subject.

|  | DPS | UPS | IPS | 4kT |
| --- | --- | --- | --- | --- |
| subject 1 | 0.32 | 0.24 | 0.21 | 0.11 |
| subject 2 | 0.45 | 0.34 | 0.26 | 0.16 |
| subject 3 | 0.41 | 0.34 | 0.24 | 0.15 |
| subject 4 | 0.29 | 0.27 | 0.21 | 0.17 |


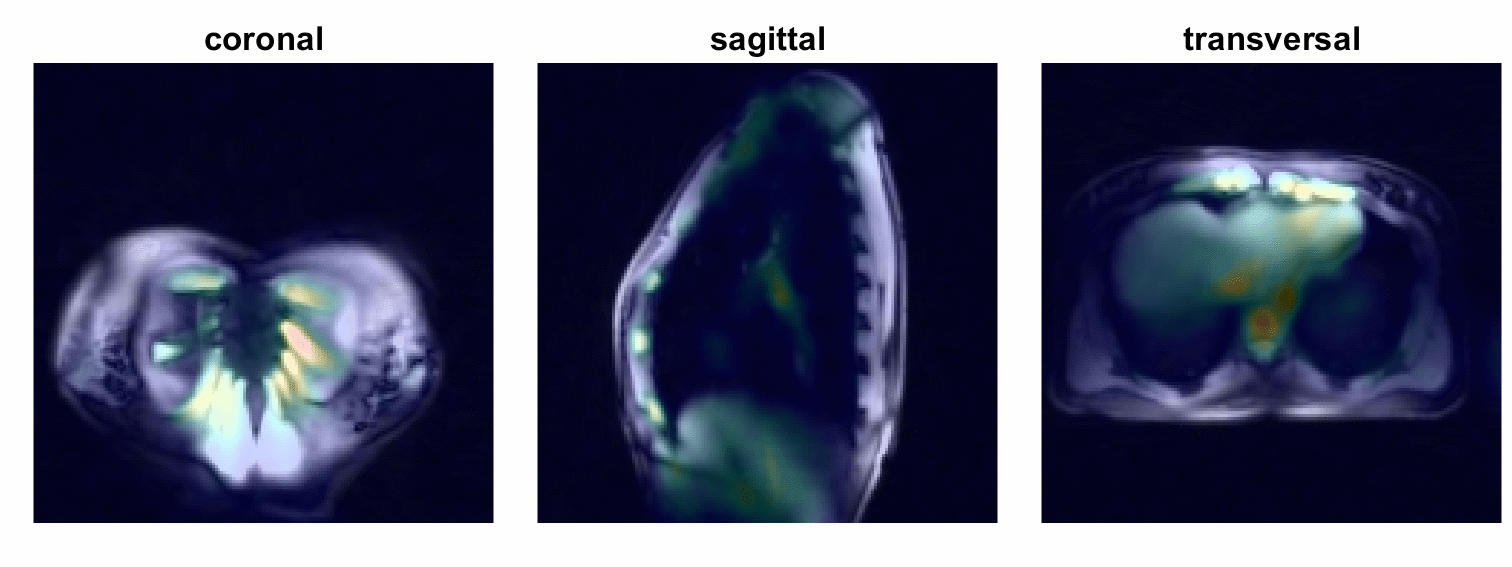


Figure S4: Animated overlay of ^23^Na and ^1^H images (subject 1) acquired with the interleaved ^23^Na/^1^H pTx sequence using the UPS for ^1^H excitation. Due to the interleaved ^23^Na/^1^H acquisition scheme, the corresponding ^23^Na and ^1^H images are aligned without the need for image co-registration.
